# Telomerase Regulation from Beginning to the End

**DOI:** 10.3390/genes7090064

**Published:** 2016-09-14

**Authors:** Deanna Elise MacNeil, Hélène Jeanne Bensoussan, Chantal Autexier

**Affiliations:** 1 Bloomfield Centre for Research in Aging, Lady Davis Institute for Medical Research, Jewish General Hospital, 3755 Côte Ste-Catherine Road, Montréal, QC H3T 1E2, Canada; deanna.macneil@mail.mcgill.ca; (D.E.M.); helene.bensoussan@mail.mcgill.ca (H.J.B.); 2 Room M-29, Department of Anatomy and Cell Biology, McGill University, 3640 University Street, Montréal, QC H3A 0C7, Canada; 3 Department of Experimental Medicine, McGill University, 1110 Pins Avenue West, Room 101, Montréal, QC H3A 1A3, Canada

**Keywords:** telomerase, telomere, H/ACA ribonucleoprotein, small nucleolar RNA, small Cajal body RNA, spliceosome, exosome

## Abstract

The vast body of literature regarding human telomere maintenance is a true testament to the importance of understanding telomere regulation in both normal and diseased states. In this review, our goal was simple: tell the telomerase story from the biogenesis of its parts to its maturity as a complex and function at its site of action, emphasizing new developments and how they contribute to the foundational knowledge of telomerase and telomere biology.

## 1. Introduction

Eukaryotic organisms with linear chromosomes face the molecular dilemma of protecting chromosomal ends from being recognized as DNA breaks, while also preventing loss of genomic information through progressive chromosome shortening caused by the semi-conservative replication of DNA [1]. In humans, these DNA ends, or telomeres are 5–15 kb of double stranded 5′-TTAGGG-3′/3′CCCTAA-5′ sequence repeats which terminate in a single stranded 3′ G-rich overhang of about 50–300 bases [2,3,4]. Telomeric integrity has implications in both cancer and aging, as telomere attrition serves as a key checkpoint in the control of cell proliferation by triggering replicative senescence. There are two broadly defined mechanisms of telomere maintenance in humans: telomerase-mediated maintenance and ALT (alternative lengthening of telomeres). However, the complexity of each of these mechanisms becomes more evident with every new publication in the field of telomere biology. Approximately 80% of cancers are immortalized by constitutive activation of telomerase to maintain telomeres throughout rapid cellular proliferation [5]. Additionally, defects in telomerase and other telomere maintenance components cause premature aging syndromes like dyskeratosis congenita (DC), due to progressive telomere shortening and subsequent proliferative blocks [6]. As such, greater knowledge of telomerase regulation and its contribution to telomere homeostasis will contribute to our understanding of human disease and natural cellular processes alike.

There are a variety of telomerase components, whether considered part of the active holoenzyme or indirect regulators of telomerase function. In vitro telomerase activity can be reconstituted minimally with the catalytic subunit human telomerase reverse transcriptase (hTERT) in combination with its guide and template RNA component human telomerase RNA (hTR) [7,8,9]. However, what has been reported as essential for telomerase activity in vivo includes functions of RNA processing, proper enzymatic assembly and trafficking, and stimulation of activity at the telomeric substrate itself. In this review, our aim was to consolidate what is known about the many steps involved in telomerase-mediated telomere maintenance in mammalian cells, with a particular focus on novel, recent advances in our understanding of the processes governing telomerase. Discussion of telomerase-mediated telomere maintenance regulation in other organisms is limited to highlighting key mechanistic differences or similarities. More specifically, it is our hope that this review will serve as an up-to-date resource for examining the “big picture” of telomerase, from hTR processing to telomere synthesis.

## 2. Transcription and Processing of hTR

**Figure 1:**
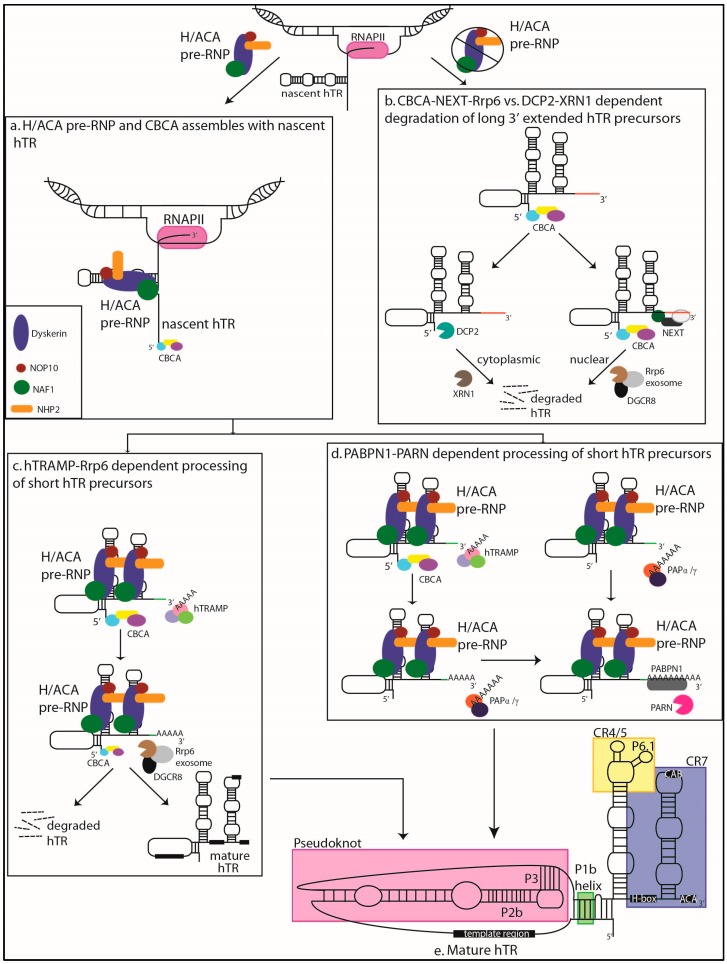
Schematic of human telomerase RNA (hTR) synthesis and processing. RNA polymerase II (RNAPII) read-through can generate 3′ extended hTR products, which need to be processed into mature hTR or degraded. (**a**) The H–ACA pre-RNP (ribonucleoprotein) complex involving dyskerin, NOP10, NHP2, and NAF1 co-transcriptionally assembles on the 3′ hairpin-hinge-hairpin-tail structure of hTR, possibly mediating RNAPII transcription termination. (**a**,**b**) The generation of shorter extended products versus longer extended products due to RNAPII read-through may be regulated by assembly of the H/ACA pre-RNP; (**b**) Defects in dyskerin–hTR interactions and RNP assembly lead to the generation of long extended hTR species, which can be exported to the cytosol for decapping mRNA 2 (DCP2)/ 5′-3′ Exoribonuclease 1 (XRN1) mediated degradation. It is possible that this export occurs in the absence of nuclear exosome targeting (NEXT) recruitment through 5′ cap-binding complex (CBCA), as NEXT is involved in the recruitment of the nucleolar Rrp6 exosome. b,**c**) The Rrp6-mediated human exosome may be involved in both maturation and degradation pathways for extended products, in conjunction with the micro-RNA processing component DGCR8. (**d**) Short extended products are targeted for processing by the canonical poly-adenylation machinery involving PAPα/γ in a poly(A) binding protein nuclear 1 (PABPN1) and polyadenosine-specific ribonuclease (PARN) dependent manner; (**c**) Shorter extended hTR species can also be targeted to the Rrp6 exosome through the addition of a shorter poly(A) tail by the Trf4/5-Air1/2-(TRAMP) complex, recruited by CBCA. The mechanism behind balancing Rrp6 exosome-mediated degradation and maturation remains inconclusive; (**d**) However, poly(A) tails added by the TRAMP complex may be extended by PAPα/γ to generate PABPN1/PARN processing targets. Given that CBCA is known to recruit TRAMP and repress PARN, the presence of CBCA at the pre-hTR species 5′ end may be relevant in mediating these pathways; (**e**) The structural domains of mature hTR are denoted in coloured boxes: the pseudoknot region in pink containing the template, P2b and P3 regions; P1b helix in green; CR4/5 domain in yellow containing the P6.1 stem-loop; and the CR7 domain in blue containing both the H/ACA and Cajal body (CAB) boxes.

The mature RNA guide and template component of telomerase is a highly divergent transcript, varying across species with respect to sequence, length, structure, and synthesis [10,11,12]. In humans, mature telomerase RNA is a non-polyadenylated 451 nucleotide (nt) product of RNA polymerase II (RNAPII) transcription [11] (Figure 1e). In contrast to fungal TRs which structurally resemble small nuclear RNAs (snRNAs) [13,14,15], hTR contains a conserved H/ACA-box motif at its 3′ terminus similar to that of the so-named H/ACA small nucleolar (sno-) and small Cajal body (CB) specific (sca-) RNAs and which is discussed in further detail in Section 3 [16,17,18]. This structural motif fosters interactions with a wide variety of processing and ribonucleoprotein (RNP) assembly factors, including co-transcriptional association with the H/ACA pre-RNP complex components dyskerin, NOP10, NHP2, and NAF1 (Figure 1a) [19]. While not needed for telomerase activity in vitro, the H/ACA domain of hTR is required for its in vivo accumulation and telomerase biogenesis [16,20]. Unlike most human sno/scaRNAs which are exonucleolytically processed from spliced pre-messenger RNA (mRNA) introns [21], hTR synthesis is more similar to that of yeast snoRNAs which is an independent transcriptional unit of RNAPII [11,22,23]. Furthermore, as is the case for many RNAPII transcripts [24], hTR is initially a 3′ extended product which is processed post-transcriptionally into a mature form [25]. In fact, both 3′ extended and polyadenylated hTR species have been previously reported [25,26], though the biological role of these immature species has remained unexamined. Recent advances have been made towards elucidating the mechanisms managing this processing and quality control of hTR maturation and accumulation (see Figure 1 for schematic compilation). As such, it is becoming increasingly clear that numerous mechanisms contribute to TR divergence.

### 2.1. hTR and the Spliceosome

Two main mechanisms of TR processing have been reported in yeast. One seemingly common pathway of 3′ TR maturation among fungi resembles a removal mechanism for improper splicing products [27,28]. However, Tseng et al. provided evidence against the involvement of the spliceosome in hTR 3′ processing, demonstrating that while the spliceosomal inhibitor isoginkgetin contributed to accumulation of 3′ extended hTR, another spliceosome inhibitor spliceostatin A had no effect. Furthermore, modification of potential 5′ splice sites downstream of the mature hTR 3′ terminus did not affect the levels of accumulated long products. Rather, they found the effects of isoginkgetin on hTR processing resembled those of exosome inhibition [29].

### 2.2. hTR and the Exosome

Interestingly, another previously reported mechanism of TR processing in yeast relies on the nuclear exosome for trimming or degradation of precursors and extended products [30,31,32]. This pathway reported in *Saccharomyces cerevisiae* depends on transcriptional termination mediated by the Nrd1-Nab3-Sen1 (NNS) complex, which recruits the non-canonical nuclear polyadenylation Trf4/5-Air1/2-Mtr4 (TRAMP) complex. Another polyadenylation-dependent processing pathway resembling mRNA 3′ maturation was reported for snoRNAs in *Saccharomyces pombe*, involving the canonical polyadenylation polymerase Pla1 and the poly(A)-binding protein Pab2 [33,34]. Ultimately, both of these pathways recruit the nuclear exosome involving Rrp6 as the exonuclease subunit for RNA processing or degradation [35]. With the exception of the NNS complex which does not appear to be conserved in mammals [36], human analogues of the key components in these pathways are beginning to be examined for hTR maturation. A role for the nucleolar human Rrp6-exosome in quality control of hTR-extended products has been proposed by several groups [29,37,38,39], and this involvement is at least in part dependent upon a non-canonical role of the micro-RNA processing component DiGeorge critical region 8 (DGCR8) (Figure 1b,c) [37]. However, it is unclear whether the exosome plays a part in the maturation or solely the degradation of hTR. Tseng et al. reported that inhibition and knockdown of the exosome lead to accumulation of long hTR precursors/3′ extended hTR transcripts (red extension in Figure 1b), and that exosomal degradation of these products is dependent upon the 5′ cap-binding complex (CBCA) and its recruitment of the nuclear exosome targeting (NEXT) complex [29]. The accumulation of 3′ extended hTR species upon depletion of any of these three complexes (Rrp6, CBCA, or NEXT) suggests a functional conservation between mammalian NEXT and the yeast NNS transcription termination complexes, as the CBCA in *S. cerevisiae* is necessary for co-transcriptional recruitment of the NNS to promote proper termination and 3′ end processing of RNAPII transcripts [29,40].

In contrast to long product processing, shorter precursors (green extension in Figure 1c,d) may be processed or degraded by the exosome following polyadenylation by the human TRAMP complex, which can also be recruited by the CBCA (Figure 1c) [29,35]. It is possible that the involvement of NEXT versus TRAMP depends on co-transcriptional assembly of the precursor hTR with H/ACA pre-RNP components. In particular, precursors which do not efficiently assemble with the pre-RNP may be more likely to assemble with the NEXT complex [29], given that they may be subject to excessive RNAPII read-through (Figure 1b). The dyskerin homologue in *S. cerevisiae* (Cbf5p) is required at snoRNA genes during transcription to prevent RNAPII read-through and promote efficient transcription termination [41]. Indeed, Tseng et al. suggested that coupling co-transcriptional pre-RNP assembly to processing of hTR would act as an efficient quality control mechanism, similar to that observed for snRNAs [29,42]. Additionally, the negative effects of deficiencies in dyskerin or hTR’s inability to assemble with the RNP have been recently attributed to exosome-dependent quality control of hTR [39]. Specifically, reduction of hTR levels and telomerase activity caused by dyskerin depletion or mutant hTR which disrupt RNP biogenesis can be rescued by knockdown of Rrp6. It was reported that Rrp6-mediated decay of hTR is enhanced by polyadenylation by the human TRAMP complex poly(A) polymerase (human poly(A) RNA polymerase D5 (PAPD5), also known as Trf4-2, homologue of Trf4) [39]. There is also evidence of a cytoplasmic 5′-3′ decay mechanism for dysfunctional hTR precursors, involving decapping mRNA 2 (DCP2) which canonically removes the CBCA from faulty mRNA transcripts exported to the cytoplasm for targeted degradation by Xrn1 (5′-3′ Exoribonuclease 1) (Figure 1b). This mechanism was reported to function independently of exosome-mediated decay [39]. It will be interesting to examine the nature of the 3′ extensions for these cytoplasmically exported hTR species, which seem to result from a lack of dyskerin assembly. A model of TRAMP-mediated exosomal degradation of extended hTR products was also proposed by Nguyen et al. in which decay and processing were found to be separate pathways in competition [38]. It was speculated that long 3′ extended products are non-functional hTR species which result from improper transcription termination and RNAPII read-through (Figure 1b), though the mechanism of hTR transcription termination has yet to be reported [38]. As such, involvement in the maturation process cannot yet be excluded for the exosome. However, it is clear that a balance between maturation and degradation of 3′ extended products must be maintained for telomerase function, and that the nucleolar exosome is a key component in this process.

### 2.3. A Processing Role for PARN

As previously stated, involvement of the canonical mRNA 3′ maturation pathway in *S. pombe* snoRNA processing was reported to involve the exosome. This mechanism is dependent upon the polyadenylation polymerase Pla1 and the nuclear poly(A)-binding protein Pab2 [33,34]. Notably, the human homologue of Pab2, poly(A) binding protein nuclear 1 (PABPN1) was recently implicated in hTR 3′ maturation through a polyadenylation dependent pathway (Figure 1d) [38]. In contrast to the exosome-driven snoRNA maturation mechanism in fission yeast, it seems that PARN (polyadenosine-specific ribonuclease) is the key nuclease for trimming of polyadenylated precursor hTR (Figure 1d) [38,43]. In fact, a competing or antagonistic role has been suggested for exosomal decay versus PARN-mediated processing of hTR precursors [29,38,39]. Nguyen et al. reported that depletion of either PABPN1 or PARN contributes to increased cellular amounts of polyadenylated and 3′ extended hTR species, and a reduction of mature hTR. In addition, knockdown of the canonical polyadenylation Pla1 human homologues PAPα/γ led to a reduction of hTR, and approximately half of PABPN1-associated poly(A) hTR species were reported to have long (>15 nt) poly(A) tails typical of RNAs generated by canonical polyadenylation polymerases. Meanwhile, depletion of components from the TRAMP complex led to an increased accumulation of mature hTR, presumably due to a lack of exosome-mediated decay, thus demonstrating the possible competition between exosome-mediated degradation and PARN-mediated trimming (Figure 1c,d) [38]. Furthermore, Shukla et al. have also suggested competition between PARN and the exosome for poly(A) hTR processing. However, it was reported that the poly(A) substrates of these nucleases are products of TRAMP polyadenylation [39]. While this is in disagreement with the proposal of canonical polyadenylation by PAPα/γ for proper hTR maturation [38], the ability of PARN to remove TRAMP polyadenylation products from hTR was also reported by Tseng et al., who proposed that PARN activity would be favored when hTR is 5′mono-methyl guanosine capped, which negatively regulates CBCA[29]. It was suggested that, though TRAMP-synthesized poly(A) tails are traditionally short (4–5 nt) (Figure 1c,d), it is possible for canonical polyadenylation polymerases to extend TRAMP products in order to foster PABPN1 interaction and PARN recruitment (Figure 1d) [29]. As such, the apparent complexity of PARN-mediated hTR processing will need further and thorough examination in the future.

Importantly, it is evident that both PABPN1 and PARN are essential for accumulation of mature hTR and effective telomere maintenance. Consistent with the importance of PARN in telomere maintenance, PARN was recently implicated in disease pathology of the premature aging diseases DC and idiopathic pulmonary fibrosis [44,45,46]. While it was suggested that PARN dysfunction could affect a number of gene products implicated in DC, PABPN1 depletion was previously reported to have no effect on the mRNA levels of many telomere maintenance gene products, nor on the normal expression of approximately 96% of polyadenylated mRNAs [47]. Therefore, it is likely that the observed telomeric effects of PABPN1 deficiency, and consequently of PARN are truly specific to hTR processing. Interestingly, it was reported that PABPN1 interacts with both dyskerin and hTERT in an RNA-dependent manner, suggesting that co-transcriptional hTR assembly for each of these components is possible [38]. RNP assembly of hTR will be discussed next.

## 3. Assembly and Localization of H–ACA Ribonucleoprotein Complex

### 3.1. The Four Musketeers on the H/ACA Motif

As previously mentioned, hTR contains the H/ACA-box motif, which is a conserved secondary structure comprised of two hairpins separated by a single stranded H-box (5′-ANANNA-3′ sequence where N is any nucleotide), ending in a single stranded 3′ tail-encoded ACA [48]. DC patients with a mutant hTR H-box display short telomeres and reduced telomerase activity [49]. It is known that this motif is necessary for the proper assembly of the telomerase complex and for its trafficking and activity [23,25,50]. This motif is found in evolutionarily conserved H/ACA sno- and sca-RNAs that are essential to target ribosomal RNAs (rRNAs) and spliceosomal snRNAs, respectively, for the post-transcriptional pseudouridine modification [51,52]. These H/ACA sno/scaRNAs guide the various functions of H/ACA RNP complexes. Interestingly, these highly conserved RNAs assemble with four evolutionarily conserved scaffold proteins upon maturation: the pseudouridine synthase dyskerin; RNA interacting components NHP2 and NOP10; and a pseudouridylation-catalysis enhancer GAR1 [53].

Variations in the genes encoding these core proteins have been characterized as DC-causing mutations. The X-linked form of this disease is due to mutations in *DKC1* which encodes dyskerin. X-DC-causing mutations concentrate in regions encoding the pseudouridine synthase and archaeosine transglycosylase (PUA) RNA binding domain or at the N-terminus of the protein [54]. Mutations in *NOLA3* (encoding NOP10) have been linked to the autosomal recessive form of DC, including one that encodes a R34W substitution in a conserved residue thought to be involved in RNA interactions [55]. Five mutations in the NHP2-encoding *NOLA2* gene have also been identified in a cohort of autosomal recessive-DC patients [56]. On the other hand, no *GAR1* mutations have been identified to date or linked to DC [57]. The identification of DC-associated and causative genetic abnormalities linked to telomerase activity initiated a wave of research oriented towards characterizing the relationship between the main telomerase catalytic components hTERT and hTR and the evolutionary conserved H/ACA RNP proteins.

**Figure 2:**
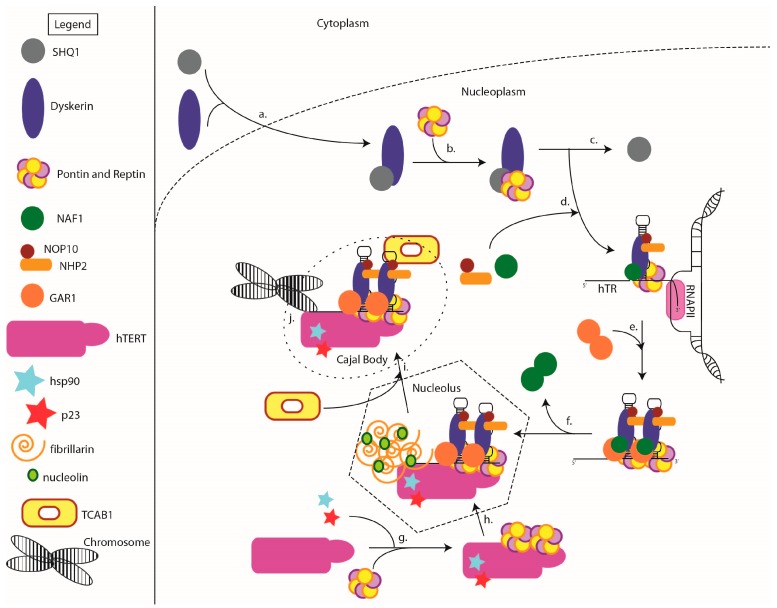
Assembly and Localization of H/ACA Ribonucleoprotein Complex. (**a**) SHQ1 binds to free dyskerin in the cytoplasm and translocates to the nucleus; (**b**) The reptin/pontin hexamer binds the dyskerin–SHQ1 complex directly to both dyskerin and SHQ1; (**c**) promoting SHQ1 removal from dyskerin; (**d**) This promotes the recruitment of the four H/ACA RNP proteins, dyskerin, NAF1, Nop10 and NHP2 to the nascent hTR; (**e**) GAR1 displaces NAF1 through the formation of a heterodimer which in turn forms a pool of NAF1 homodimers; (**f**,**g**) The mature hTR gets recruited to the nucleolus where it assembles with hTERT which was previously processed by hsp90, p23 and the reptin/pontin hexamer; (**h**) This allows hTERT recruitment to the nucleolus to form a mature telomerase complex while bound to fibrillarin and nucleolin with the hTR; (**i**) Telomere Cajal body protein 1 (TCAB1) recognizes the Cajal body (CAB) box of the hTR in the mature telomerase complex and recruits it to the Cajal body; (**j**) In S-phase, the Cajal body will colocalize with telomeres and facilitate the recruitment of the mature telomerase complex to the telomeres.

A closer look at the assembly of hTR with these core proteins at the H/ACA motif reveals a similar interaction to the one observed in sno/scaRNAs (see Figure 2). Two full RNP complexes bind to the hTR, one at each hairpin [58]. Dyskerin and NOP10 interact with hTR while GAR1 and NHP2 are recruited through protein–protein interactions to dyskerin and NOP10, respectively [19,59] (Figure 2d). Although hTR contains a canonical H/ACA motif, it differs from typical human H/ACA sno/scaRNAs as it has not been reported to be involved in pseudouridylation or any RNA post-transcriptional modification activities [19,52]. The role of its 3′ H/ACA motif seems to be focused on the regulation of its biological stability and function with respect to telomerase. While the structure of the mature RNP has been accurately described, there are unanswered questions regarding the process of its assembly. One important question is whether all the RNP components assemble as a tetrameric complex prior to being recruited to the RNA or if they assemble with one another while associating with a nascent RNA. The core complex formation is necessary for hTR stability and accumulation in vitro which was also confirmed in vivo [60,61]. The point at which GAR1 interacts with the other core components also remains somewhat of a mystery in the assembly process of the mature H/ACA RNP. While Dyskerin-NOP10-NHP2 colocalize at the site of hTR transcription, GAR1 does not [62]. Therefore, GAR1 must join the complex later to form the mature RNP (Figure 2e).

### 3.2. Assembly Factors

The complex preceding GAR1 association is known as the pre-RNP and includes the association factor nuclear assembly factor 1 (NAF1). NAF1 cannot bind hTR without the presence of the dyskerin-NOP10-NHP2 trimer [63]. Concordantly, this small protein is essential to the assembly of the pre-RNP complex with hTR and is not present in the mature RNP complex [41,62,64]. Prevalent DC-causing substitutions in dyskerin, such as A353V, do not impair the pre-RNP complex protein–protein interactions in vitro but lead to a decrease in hTR assembly with the pre-RNP complex. It can be hypothesized from these data that the H/ACA pre-RNP proteins can assemble prior to binding hTR [63]. From their findings in 2006, Darzacq et al. proposed a stepwise RNP assembly model where NAF1 binds to dyskerin in the cytoplasm to stabilize the protein, allowing for the recruitment of the other components (NOP10 and NHP2). Furthermore, NHP2 can only associate with the pre-RNP tetramer in the presence of NOP10. Therefore, NOP10-NHP2 likely bind to dyskerin as a heterodimer. Then, NAF1 can act as a nucleolar shuttle, bringing an inactive precursor complex to the nascent hTR to form the pre-RNP complex (Figure 2e,f). Most recently, mutations in the gene encoding NAF1 were identified in pulmonary fibrosis-emphysema patients, causing decreased telomerase RNA accumulation and telomere shortening [65]. While these mutations also affect levels of other H/ACA RNAs, no rRNA pseudouridylation defects or ribosomal pathology is observed in first generation mice with these mutations. This newly implicates NAF1 haploinsufficiency specifically in telomere maintenance syndromes and is consistent with an important role of NAF1 in telomerase H/ACA RNP assembly and hTR stability [65].

The above model has however been revisited with the identification of a novel nuclear assembly factor, SHQ1 [66,67]. Grozdanov et al. show that SHQ1-binding domain mutants of dyskerin are associated with DC, resulting in lower levels of dyskerin and hTR instability [68]. Further research has led to a more in-depth characterization of SHQ1. While SHQ1 co-immunoprecipitates with free dyskerin in the nucleus, SHQ1 does not associate with dyskerin when dyskerin is assembled in an H–ACA RNP complex. Moreover, SHQ1 has never been found to simultaneously interact with dyskerin bound to NAF1 [67]. This HSP90-like chaperone seems to play a major role in the regulation of free dyskerin levels, protecting the protein from degradation through transient interactions prior to the H/ACA pre-RNP assembly. Moreover, the C-terminal SHQ1-Specific Domain (SSD) of SHQ1 was shown to mimic RNA-interaction patterns at the dyskerin PUA domain and compete with binding of RNAs to dyskerin [66,67,69,70]. It was later shown by structural and biochemical analysis that SHQ1 binding to dyskerin does not prevent NAF1, NOP10 and NHP2 from assembling in vitro [69]. Rather, its RNA mimicking function may indicate that it would be dislodged from dyskerin by the RNA component as they compete for the dyskerin RNA binding site [69]. Additionally, endogenous SHQ1 localizes to the cytoplasm and nucleoplasm but is excluded from the nucleoli and the GAR1-rich CB (Figure 2a–c). All these findings support a stepwise-regulated assembly of the H/ACA RNPs in the nucleolus starting with SHQ1-bound dyskerin shuttling to the nucleoplasm where SHQ1 is displaced by an RNA–dyskerin interaction prior to dyskerin’s assembly with NAF1, NOP10, and NHP2 (Figure 2c,d). This model contradicts the hypothesis that the pre-RNP tetramer assembles in the cytoplasm.

Interestingly, NAF1 is also excluded from the CB and nucleolus [64]. Once the pre-RNP is assembled, NAF1 must be substituted by GAR1 to form a mature complex (Figure 2e,f). Leulliot et al. found that GAR1 substitutes NAF1 binding to dyskerin in a competitive manner through the formation of a NAF1-GAR1 heterodimer. Using crystallography (PBD ID 2V3M), NAF1 was shown to form a dimer in solution, and this homodimer is disrupted by dyskerin interactions [71]. Moreover, monomeric NAF1 has little affinity for dyskerin and importantly, the NAF1-binding interface of dyskerin is homologous to the GAR1-binding domain. Together, GAR1 and NAF1 create a heterodimer which reduces NAF1 affinity for dyskerin and separates it from the complex leaving the site available for GAR1 binding. The higher affinity of GAR1 to dyskerin allows for the displacement of NAF1 in solution, which is forced to form a homodimer for stability [71]. The substitution of GAR1 for NAF1 is a key distinguishing characteristic between an active mature RNP complex and a pre-RNP complex [71]. A recent study in Archaea by Wang et al. shows that GAR1 binding to dyskerin is essential for the proper placement of the target RNA component in the mature H/ACA RNP pseudouridylation pocket, as well as for its release post-pseudouridine modification [53]. However, the localization of GAR1 recruitment remains unclear. For example, the mature hTR RNP may assemble in the nucleolus during H/ACA RNA processing, or perhaps GAR1 joins the complex at a later maturation step in the subnuclear membrane-free organelles referred to as CBs [61]. This localization of hTR to the CBs will be discussed next.

### 3.3. Localization to the CB

After its assembly into a pre-RNP complex and following processing in the nucleolus, hTR localizes to the CB where it is found as a mature RNP complex bound to GAR1 [17,72] (Figure 2i). CBs are subnuclear dynamic sites which are also referred to as coiled bodies. These sites are typically where snRNAs are synthesized and processed [61]. One such example of snRNA processing is pseudouridine modification by H/ACA RNPs containing scaRNAs as guides. It is certain that mature H/ACA RNPs guided by scaRNAs localize to CBs at some point during their lifetime. An experiment following dyskerin expression in a time-dependent manner confirms an initial accumulation in the nucleolus and subsequent localization to the coiled bodies [73]. Though there has been no reported evidence of a pseudouridylation target for hTR, its CB localization is well established. With respect to human telomerase, a better understanding of this trafficking mechanism accompanied the discovery of the CAB-box contained in the 3′ hairpin of the hTR H/ACA motif, which is a CB-specific localization sequence [74]. Mutating this conserved sequence impairs hTR localization to the CB and can cause DC, exemplified by the C408G mutant hTR identified in autosomal DC patients [25,49,75]. A WD40 protein named Telomere Cajal body protein 1/WD repeat-containing protein 79 (TCAB1/WDR79) was identified to bind to the CAB-box in scaRNAs to bring them to the CBs [50,76]. CAB-box sequence mutants prevent TCAB1 from binding to the RNA and impair its recruitment to the CBs [77,78]. Inversely, depletion of TCAB1 leads to a G1 cell cycle arrest and prevents the recruitment of telomerase to telomeres [79]. Therefore, this protein acts as a scaffold for the mature hTR-H/ACA RNP to localize to the CBs.

However, the biological role of this recruitment remains unclear as recent studies have shown that these subnuclear bodies are not essential for telomerase activity in vivo in human cells [25,80], though CBs have been reported to colocalize with telomeres during S-phase when telomerase is functional [50]. In cells depleted of the key CB scaffolding protein coilin, hTR assembly with telomerase is not affected and the telomerase holoenzyme remains functional [77,80] (Figure 2j). However, variations in these results were observed depending on levels of telomerase expression. Although hTR is expressed and is capable of assembling into an active telomerase complex with the other components, the ability of the telomerase holoenzyme to repeatedly translocate and synthesize telomeric DNA (known as repeat addition processivity (RAP)) is reduced in the absence of CBs [77]. Furthermore, hTR was reported to colocalize with CBs and telomeres when expressed in mouse cells, though mouse telomerase regulation appears independent of CBs [81]. It was recently shown that the CB element coilin associates with a NAF1-free dyskerin in an hTR-dependent manner and that coilin expression actually decreases dyskerin-hTR assembly, acting as an inhibitor of telomerase assembly or activator of its disassembly [82]. On the other hand, in cancer cell lines, the overexpression of telomerase components was shown to promote efficient recruitment to telomeres in the absence of CBs. These results suggest that recruitment of the mature complex to CBs facilitates telomere encounters by the telomerase holoenzyme under normal biological conditions, but when telomerase is no longer limiting overexpression, these organelles can be bypassed by functional telomerase complexes. Interestingly, Vogan et al. [83] recently reported that maintenance of telomere length homeostasis does not require localization of hTR to CBs, as knockout of either TCAB1 or coilin has no effect on the telomeric function of a minimal hTR species when hTERT is overexpressed. Furthermore, Vogan et al. observe that telomere length homeostasis is maintained in TCAB1 or coilin knockout cells expressing endogenous telomerase levels, albeit at a shorter stable length than non-knockout parental cells [83]. As telomerase is found in low levels in normal human cells, localizing to the telomeres is likely to need chaperoning mechanisms such as CB accumulation [80,84].

### 3.4. Cell Cycle Dependent Assembly and Localization

CBs are mobile structures, and therefore have the capacity to move the associated telomerase holoenzyme to its target when needed [85]. This movement is proven to be cell-cycle dependent with a two-fold increase of hTR colocalization at the telomeres and CBs during S-phase [86,87]. However, Vogan et al. recently showed that general hTR and telomerase assembly remain constant throughout cell cycle progression. In fact, TCAB1 association to the CAB-box of hTR seems to be the RNP maturation step which is regulated by the cell-cycle. Association of TCAB1 to hTR peaks in S-phase and is diminished in M phase. Therefore, TCAB1’s role may not only be to regulate the trafficking of telomerase to the CBs, but also to modulate access of active telomerase to the telomeres [88]. Observing the cell-cycle regulation of the mature complex provides insights on the stepwise assembly of the pre-RNP complex to the telomerase catalytic subunit hTERT. Tomlinson et al. show that hTR accumulates first at the CBs in G1 and beginning of S-phase, unassociated to hTERT. They show that hTR and hTERT are only found to colocalize in S-phase, suggesting that hTERT assembles with hTR at the CBs, at which point the telomerase holoenzyme becomes mature and active [87]. Interactions between TCAB1 and dyskerin, hTR, and hTERT have also been previously reported, providing additional evidence that the mature telomerase holoenzyme is assembled once located at the CBs [50]. In further studies, the same group demonstrated that hTERT is essential for hTR localization to the CBs during S-phase in cancer cell lines. These two findings seem contradictory as the second implies that hTR association to hTERT is essential for its recruitment to CBs. The maturation pathway is partially clarified by Ji Hoon Lee et al. who reported that hTERT associates with the pre-RNP in the fibrillarin component of the nucleolus while bound to nucleolin, prior to the recruitment of the mature RNP to the CBs by TCAB1 where the telomerase holoenzyme becomes catalytically active [89] (Figure 2g–i). This is an excellent example of the importance of chaperones in hTERT localization and RNP assembly.

### 3.5. Chaperones: Small Particles, Essential Roles

In vivo, additional factors besides hTR and hTERT are indispensable for the activity and recruitment of the telomerase holoenzyme to the telomeres. One such co-factor for telomerase is HSP90, a highly conserved molecular chaperone involved in cell cycle regulation, chromosome integrity and other signaling pathways [90,91]. This protein is involved in both promoting hTERT binding to the hTR component as well as telomerase DNA binding [91,92,93]. Chiu et al. show that the inhibition of HSP90 leads to the inhibition of telomerase activity and an increase in apoptosis [94]. This effect is not only due to protein–protein binding of the chaperone to hTERT but also HSP90 binding to the hTERT promoter, which will be discussed in more detail in Section 4.1 [92,95]. Importantly, it was recently reported that while HSP90 along with other heat shock complex components (HSP70, p60/Hop, HSP40, and p23) expressed in *E. coli* with hTERT and hTR can reconstitute telomerase activity, inhibition of HSP90 after telomerase assembly does not affect activity [96]. Furthermore, the foldosome component p23 which forms a complex with HSP90 is also known to be involved in telomerase regulation [92,97,98]. Both chaperones associate stably with the N-terminus of hTERT and, interestingly, remain associated with active telomerase after proper folding of hTERT has taken place. It is fascinating that these chaperones, in addition to taking part in a more canonical role of complex assembly, also seem to regulate telomerase activity post-assembly [98] (Figure 2g–j).

Last but not least, two ATPases Associated with diverse cellular Activities (AAA+ ATPases) pontin and reptin are key elements in the formation and function of a mature RNP complex. These two AAA+ ATPases are part of many transcriptional regulation and chromatin remodeling complexes, and contribute to cellular growth regulation and DNA damage repair [99]. The growing interest in these yeast-helicase homologues arose because they were found to be overexpressed in a myriad of cancers. It was reported that overexpression of either protein increases cell proliferation [100,101]. Venteicher et al. conducted the first research linking pontin and reptin to the assembly and function of the mature telomerase holoenzyme, showing that the complex not only binds directly to hTERT but also to hTR and dyskerin in vitro (Figure 2b,d and g). Additionally, they demonstrated that the assembly and stabilization of the mature RNP is ATP-dependent and cell cycle regulated with a peak of the AAA+ ATPases recruitment in S-phase [102]. Further experiments uncovered that SHQ1 removal favours the formation of the pre-RNP complex in a reptin/pontin dependent manner [99] (Figure 2c). The role of the essential non-dyskerin-binding CS domain of SHQ1 in the assembly process was also puzzling to scientists. Machado-Pinilla et al. show that both reptin and pontin, individually or together, are capable of binding both the CS domain of SHQ1 and the highly charged C-terminal tail of dyskerin (Figure 2b). The reptin and pontin structures solved by cryo-electron microscopy show that they assemble as a hexameric ring[103], leading to the proposed model that the ring catches the dyskerin C-terminal tail and stabilizes it to facilitate SHQ1 removal through CS domain pulling. Moreover, reptin and pontin have been linked to the posttranslational modification of small ubiquitin-like modifier (SUMO) conjugation (SUMOylation), as pontin-chromatin complexes are associated with the SUMO-conjugating enzyme ubiquitin-conjugating enzyme 9 (Ubc9) and reptin–chromatin complexes are associated with the deSUMOylation enzyme sentrin-specific peptidase 1 (SENP1) [104,105]. Interestingly, dyskerin is a multiple-site target for SUMOylation as well [106]. Moreover, dyskerin SUMOylation contributes to hTR stability and accumulation, telomerase activity and telomere maintenance. Indeed, DC-causing mutations have been found to coincide with regions encoding some SUMOylation sites in dyskerin. It would be interesting to test if these mechanisms are correlated and if dyskerin SUMOylation is altered upon pontin/reptin depletion [106,107]. Just as the aforementioned components of the telomerase RNP are highly regulated, hTERT must also be processed and posttranslationally modified prior to its participation in telomere maintenance, which will be discussed below in Section 4.

### 3.6. Association of hTR and hTERT

As the hTERT-hTR interaction domains have already been thoroughly reviewed recently[108], they will be summarized here only briefly. Both the catalytic subunit and RNA template contain critical domains for their association. hTR contains the conserved regions 4 and 5 (CR4/5) domain which is essential for hTR binding to hTERT [109] (Figure 1e). One of the main regions regulating binding in this domain is the P6.1 loop [110]. Additionally, hTERT contains a N-terminal domain that includes two conserved regions: the TERT Essential N-terminal domain (TEN) domain and the telomerase RNA binding domain (TRBD) which are critical for hTR-hTERT association [111,112,113,114,115,116]. Moreover, the pseudoknot region of hTR fosters hTERT interaction near the template region of the RNA, allowing proper positioning of the template and telomeric substrate for reverse transcription and DNA synthesis [117]. The CR4/5 domain displays higher affinity for hTERT than the pseudoknot [115,118]. However, the pseudoknot is important to stabilize hTERT-free hTR in vivo, but not in vitro [115,119]. Disease-associated mutants of the conserved P2b/P3 region contained in the pseudoknot lead to hTR destabilization [115]. Zemora et al. also provided evidence that hTERT recruitment leads to an hTR conformational change through its pseudoknot that exposes its template and allows CR4/5 binding [115]. Although the domains regulating the interaction and assembly of hTERT and hTR are well characterized, the site of their assembly and the mechanisms regulating it remain unclear.

## 4. hTERT Regulation

The regulation of telomerase activity is most commonly linked to the regulation of hTERT, primarily at the level of transcription but also by posttranslational modification. This has implications in both cellular development and carcinogenesis [120,121], as telomerase activity and thus hTERT expression is needed for replicative longevity. Proper regulation of the hTERT promoter and subsequent hTERT gene transcription is known to be important for maintaining differential basal telomerase activities between cell types [120,122]. Indeed, some of the most commonly occurring non-coding mutations in human cancers are those within the hTERT promoter [123,124,125,126,127,128]. Furthermore, there have been many reports of phosphorylation and ubiquitination of hTERT [120], though a greater understanding of the mechanisms and importance of such modifications is still emerging. It has been postulated that as regulation of hTERT transcription is key for control of telomerase activity, posttranslational regulation is crucial for its adjustment [120,129]. As such, this section will focus on recent advances made in elucidating the regulation of hTERT transcription and posttranslational modifications, and how these recent advances fit into the ever-evolving knowledge of hTERT regulation.

### 4.1. hTERT Transcription

In addition to the aforementioned involvement of HSP90 in telomerase assembly and activity, this molecular chaperone was reported to interact with the hTERT promoter, regulating telomerase at the transcriptional level. Specifically, HSP90 was found to be present at the hTERT promoter in human immortalized and cancer cells, while absent from normal senescing cells which do not express telomerase [95]. It was reported that HSP90 function is required for promoter activity and transcription of the hTERT gene. Indeed, chemical inhibition of HSP90 leads to a reduction in hTERT mRNA and telomerase activity, as well as HSP90 interaction with the hTERT promoter, even in the absence of new protein synthesis [95]. Interestingly, the promoter regions with which HSP90 were reported to interact coincide with binding sites for other transcription factors (TF), including Sp1 which canonically binds GC-boxes [95]. These are especially important regions for the transcription of genes which lack a TATA-box in their promoter, like that of hTERT [130,131]. There are at least five GC-boxes within the hTERT promoter, and mutating these regions is detrimental to hTERT transcription [131]. While it does not appear that inhibition of HSP90 affects Sp1 interaction with the hTERT promoter [95], it is interesting that these two TFs have been previously reported to cooperate in regulating the transcription of other genes with GC-boxes within their promoters [132]. The cooperation of Sp1 with other known hTERT-regulating TFs such as c-Myc [133] has also been demonstrated to occur in a cell type-dependent manner [131]. It was recently reported that both Sp1 and related TF Sp3 are needed at the hTERT promoter for its transcription, and mutations in the GC-boxes do not affect the binding of other TFs to the promoter or its chromatin state [134]. These findings by Cheng et al. confirmed earlier reports of Sp1-family TFs involvement in activating hTERT transcription, while contrasting some reports of their roles in hTERT transcription repression. Notably, Sp1 binding was not reported to vary between cells which have differing hTERT promoter repressive states. In addition, binding of Sp1/3 does not affect H3K9 (histone 3 lysine 9) acetylation or trimethylation status, which are both indicative of no change to the chromatin environment. 

However, the dense heterochromatin-like state in which the hTERT gene usually exists must be resolved for access to the GC-boxes and promoter activity stimulation in telomerase positive cells specifically [134]. There have been several hypotheses on the mechanisms controlling differential hTERT expression [135,136,137,138], but recently Chen et al. proposed that E-box TFs, members of the Myc family, and/or USF1/2 may be important for hTERT promoter de-repression, possibly through recruitment of histone modifiers. This was suggested on the basis that Sp1 binding to the hTERT promoter alone is not sufficient for transcription initiation while these other TFs were found to be specifically enriched at the hTERT promoter in telomerase positive cells [134]. Importantly, these other TFs are also present in telomerase negative cells, so it is likely that some form of chromatin remodeling step is needed to allow access to the promoter to generate a telomerase positive setting specifically. The establishment of a nucleosome-free region known as a DNAse I hypersensitive site (DHS) in the hTERT promoter may contribute to this initial remodeling, as a positive correlation between hTERT transcription and the presence of a major DHS in the hTERT promoter was reported in telomerase positive cells, and the generation of a nucleosome-free region in the hTERT promoter is the rate limiting step in gene transcription [134]. Interestingly, the hypothesis of chromatin remodeling at the hTERT promoter in specific cellular conditions is not unique to GC-boxes. It has been reported using in vivo DNA footprinting that chromatin remodeling takes place at the estrogen response elements (EREs) in the promoter as well, specifically in cells which are ERα-positive [139]. Chromatin-state at the hTERT promoter can also be altered by the retinoic acid hormone receptor family, as repression of telomerase with retinoids can occur by decreasing H3K9 acetylation at the promoter, in concert with re-enforced repressive promoter methylation [140,141]. The cooperation of a wide variety of hormonal and non-hormonal regulators is apparent in modulation of hTERT transcription. For example, a c-Myc and hypoxia-inducible factor(HIF)-α mediated induction of hTERT expression was recently reported for the stress hormone norepinephrine, contributing to invasiveness and metastasis of ovarian cancer [142]. This transcription induction may be therapeutically targetable, for example as the phytoalexin resveratrol was demonstrated to attenuate norepinephrine-induced Src phosphorylation by protein kinase A (PKA) and subsequent HIF-α expression, thus reducing hTERT expression in ovarian cancer cell lines [143].

Notably, HIF-α and c-Myc are just two examples of oncogenic TFs involved in the regulation of hTERT transcription. Regulation mediated by ETS family TFs has also been well documented [144,145,146,147,148,149,150,151]. In fact, all cancer-associated mutations identified in the hTERT promoter have been reported to generate binding sites for ETS TFs, proximal to the transcriptional start site [124,125]. Due to the frequency of these non-coding mutations in human cancer, mutations in the hTERT promoter have been of particular interest in the field as of late. In particular, Chiba et al. [152] applied Clustered regularly interspaced short palindromic repeats (CRISPR)/Cas9 genome editing techniques to study the mechanisms of certain hTERT promoter mutations in telomerase positive human embryonic stem cells (hESCs). They examined three different mutations which generate putative ETS-binding sites (TTCCGG): -57A/C, -124C/T, and -146C/T. A modest increase in transcription of hTERT was observed for only the most commonly occurring -124C/T mutation, which was not sufficient to impact the activity of telomerase in the hESCs. Interestingly, Chiba et al. found that all three of these mutations led to a failure to silence hTERT expression upon differentiation of the hESCs into somatic cells. In somatic cells, telomerase is typically downregulated to ensure proper activation of growth control checkpoints prior to replicative senescence. The observed failure to silence hTERT expression was reported to cause an increase in telomerase activity and telomere lengthening, allowing the differentiated cells to bypass the canonical telomere-established proliferative block in somatic cells [152]. These findings suggest that hTERT promoter mutations are able to contribute to tumorigenesis through abnormally high hTERT expression in somatic tissues and telomerase activity resembling that of immortal cancer cell lines. Consistent with these findings, Xi et al. [153] reported a decrease in telomerase activity and proliferative capacity, in addition to telomere shortening upon reversion of the -124C/T mutation using CRISPR/Cas9 gene editing in a urethral cancer cell line which is normally heterozygous for the mutation. Meanwhile, introduction of the -146C/T mutation into HEK293T cells was not reported to increase telomerase activity. One possible explanation offered for this finding is that these cells already express hTERT and demonstrate telomerase activity in a wild type-promoter background, and as such a rate limiting step in hTERT transcription activation may already have been achieved in these cells by another mechanism. These findings corroborate those of Chiba et al., demonstrating that hTERT promoter mutations can stimulate transcription as well as telomerase activity allowing for the proliferative potential in cancerous cells, though these mutations are not necessary for active telomerase in all cells. It is clear that the activation and deactivation of telomerase which is mediated through transcriptional regulation of hTERT is key for maintaining control of cellular growth. However, an important additional step in regulating telomerase relies on posttranslational modification of the hTERT protein.

### 4.2. Posttranslational Modifications of hTERT

The posttranslational modifications of hTERT have been previously reviewed in detail [120,129]. In brief, it is known that phosphorylation of hTERT by both protein kinase C (PKC) [154] and Akt/protein kinase B (PKB) [155] is needed for telomerase activity, while phosphorylation by the c-Abl-kinase [156] and ubiquitination by Makorin Ring Finger Protein 1 (MKRN1) [157], C terminus of HSC70-Interacting Protein (CHIP) [158], and Hdm2 [159] E3 ligases lead to a reduction in telomerase activity. However, the mechanisms behind these pathways are still being clarified. The importance of hTERT cellular localization has been reported for ubiquitination. Proteasomal degradation of ubiquitinated hTERT occurs in the cytosol, and as such, controlling nuclear import/export of the protein is key for regulating this degradation. Indeed, one dominant negative form of hTERT functions through increased nuclear export of both itself and wild-type hTERT, leading to ubiquitination, proteasomal degradation, and subsequent telomere maintenance defects [160]. Interactions with 14-3-3 protein have also been reported to retain hTERT in the nucleus through preventing chromosomal maintenance 1 (CRM1)-mediated export, while a dominant negative form of 14-3-3 protein was reported to promote hTERT nuclear export [161]. Whether this interaction regulates hTERT strictly through retention within its organelle of action, through reducing proteasomal degradation, or through a combination of both mechanisms remains to be investigated. Interestingly, the novel telomerase-associated protein Polo-like kinase 1 (Plk1) was speculated to activate telomerase through a related but opposite mechanism [162]. Huang et al. reported that Plk1, while associated with an active telomerase complex, improves the loading of hTERT onto chromatin, presumably promoting its nuclear retention and inhibiting cytosolic ubiquitination. Overexpression of Plk1 leads to an increase in hTERT protein levels without affecting the mRNA levels, supporting the hypothesis that nuclear retention mediated through Plk1 prevents proteasomal degradation of hTERT and increases telomerase activity [162]. Though Plk1 is a kinase, it was not reported to phosphorylate hTERT, nor was the kinase activity of Plk1 necessary for telomerase association. Importantly, Plk1 typically cooperates with other kinases, requiring phosphorylation of other residues on the target prior to carrying out its own phosphorylation of the target [163,164]. Further investigation will be needed to determine if hTERT may also be a target of Plk1 kinase activity, secondary to other known phosphorylation events.

There is increasing evidence that phosphorylation of hTERT is also important for nuclear localization. Supporting earlier findings by Liu et al. of a correlation between phosphorylated hTERT, nuclear import, and active telomerase [165], Chung et al. reported that, along with a bipartite nuclear localization sequence, hTERT localization to the nucleus requires phosphorylation by Akt [166]. Among the possible mechanisms behind this phosphorylation leading to nuclear import, Chung et al. speculate that this posttranslational modification may cause a conformational switch which provides a newly available interaction site for binding partners which promote nuclear localization, like nuclear factor kappa-light-chain-enhancer of activated B cells (NF-ΚB) family member p65 [167]. Notably, it was later reported that dephosphorylation of hTERT by the phosphatase PP2A impairs the interaction between hTERT and 14-3-3 thus impeding proper nuclear localization, as well as reducing telomerase activity [168]. More recently still, a fascinating role of phosphorylation-mimicry in nuclear tethering was reported for the sphingolipid S1P [169]. hTERT was found to have a docking site for S1P at aspartate 684, and furthermore was observed to interact with the sphingolipid at the nuclear periphery in human fibroblasts. This interaction reduces MKRN1-mediated ubiquitination and proteasomal degradation of hTERT. The authors demonstrated that this interaction with S1P has redundancy with phosphorylation at serine 921, which has also been reported to stabilize hTERT against MKRN1-mediated ubiquitination. More specifically, abolishing the S1P interaction can be rescued by a phospho-mimetic substitution of serine 921 with aspartate, but not by a phospho-abolishing serine to alanine substitution. Meanwhile, the serine to alanine substitution at residue 921 did not affect nuclear localization nor the MKRN1-hTERT interaction when the S1P interaction remained intact. Taken together, the authors interpret these data to suggest an allosteric mimicry of phosphorylation at the hTERT C-terminus by S1P interaction, thus retaining nuclear localization and protection of hTERT against proteasomal degradation [169]. It is clear that cooperation between posttranslational modifications and posttranslational localization of hTERT is required for fine tuning of telomerase regulation, and the mechanisms dictating these processes are complex. Next, the recruitment of telomerase to its canonical site of action will be discussed as yet another means of controlling telomerase-mediated telomere maintenance.

## 5. Telomerase Recruitment to the Telomere

In an endogenous cellular setting, the amounts of telomerase and telomeric substrate are both insufficient for diffusion-mediated encounters to facilitate enzymatic activity. As such, active recruitment of telomerase to its substrate is a necessary regulatory step in telomere maintenance. The recruitment roles of telomere-interacting shelterin components TPP1 and POT1 have recently been reviewed in detail, as have the implications of mutations in the dissociates activities of telomerase (DAT) encoding regions in the hTERT TEN-domain and C-terminal extension (N-DAT and C-DAT, respectively) [108]. Concordantly, this section will primarily focus on other and/or more recently described telomerase recruitment mechanisms.

### 5.1. Shelterin and Telomerase Recruitment

Human telomeres are protected by a complex of six proteins known as shelterin. This complex directly recognizes telomeric DNA through the double-stranded DNA binding components telomere repeat-binding factors 1 and 2 (TRF1, TRF2), and single-stranded DNA binding protein protection of telomeres 1 (POT1). TRF1-interacting nuclear factor 2 (TIN2) and TPP1—previously known as TIN2 interacting protein (TINT1), POT1 and TIN2 organizing protein (PTOP) and POT1 interacting protein (PIP1)—bridge the double- and single-stranded DNA binding components, while the TRF2-binding protein, repressor activator protein 1 (RAP1) (discussed in Section 6.3) functions in regulating telomerase activity based on the number of telomeric DNA repeats present in a given substrate [170,171,172]. As was introduced, it has been established that TPP1 recruits telomerase to the telomere. Briefly, a group of amino acids in the N-terminal OB-fold of TPP1 referred to as the TPP1 glutamate and leucine-rich (TEL) patch directly interact with the TEN domain of hTERT [173,174,175,176]. Modifying either of these regions and abolishing the interaction prevents telomerase recruitment and telomeric maintenance [176].

Another function of the shelterin component TIN2 was recently identified that is separable from its TPP1-interacting function [177]. A TIN2 mutant identified in DC patients was reported to localize and function canonically at the telomere, with the exception of telomerase recruitment. This failed recruitment causes telomere shortening due to a lack of telomerase-mediated extension, consistent with a previous report that this mutant immunoprecipitates less active telomerase than wild-type TIN2 [178]. Mutant TIN2 was still able to anchor TPP1 at the telomere, so these findings do not rule out the possibility that the mutant TIN2 is able to mediate a TPP1-telomerase interaction. Additionally, though telomerase recruitment was reduced upon expression of the mutant TIN2, telomeres which were accessed by telomerase were extended normally [177]. This supports the possible separation of the TPP1/shelterin-mediated recruitment functions from telomerase stimulatory roles, which will be discussed in more detail below in Section 6.3. Furthermore, advances have also been made in understanding how TPP1 interacts with hTERT, which will be the focus of the following section.

### 5.2. hTERT and Telomerase Recruitment

As stated above, TPP1 directly recruits hTERT to the telomeres through the TEL patch-TEN domain interaction. Notably, hTERT also contains a region which makes it unique among reverse transcriptase enzymes: the insertion in fingers domain (IFD) located within the reverse transcriptase motifs [179]. The IFD was recently characterized to foster recruitment of hTERT to the telomere [180] in a TPP1 dependent manner [181,182]. Several hTERT variants in the IFD were examined for effects on telomerase activity, processivity, and telomere recruitment [180,181,182]. More specifically, defects in telomeric association (assessed by fluorescence in situ hybridization of hTR/telomere co-localization) and telomere binding (assessed by telomeric chromatin immunoprecipitation) were observed for several IFD variants. These defects could be rescued by TPP1 overexpression for some, but not all IFD variants, indicating that IFD mutants can render hTERT recruitment by TPP1 sub-optimal or entirely impossible [181,182]. It was speculated that these recruitment defects may be mediated through conformational changes to the TEN domain, supported by the proximity of the IFD to the TEN domain in recent cryo-EM mapping of the *Tetrahymena thermophila* TERT [183]. Recruitment defects may also be mediated through interactions of the IFD with other telomerase recruitment regulators. For instance, the telomerase inhibitor PIN2/TERF1 interacting, telomerase inhibitor 1 (PinX1) mediates telomerase recruitment and localization through interaction with hTERT [184]. PinX1 was found to be important for S-phase recruitment of telomerase to the telomeres [184]. Fascinatingly, it was recently reported that PinX1 interacts with and stabilizes the shelterin component TRF1 in an hTERT-dependent manner [185]. It is known that TRF1 undergoes proteasomal degradation when removed from the telomeres [186,187]. However, PinX1 interaction protects TRF1 from this degradation, and hTERT depletion protects TRF1 from degradation caused by PinX1 knockdown [185]. It will be interesting to see how these three components interact at the telomere, and whether the PinX1-TRF1 interaction may somehow regulate hTERT recruitment.

Notably, it was also recently reported that in order for hTERT to be effectively recruited to the telomeres in S-phase, TRF1 must vacate the telomere in an Ataxia telangiectasia and Rad3-related protein (ATR)/ ataxia telangiectasia mutated protein (ATM) dependent manner [188]. TRF1 dissociation from the telomere may be caused by its phosphorylation by ATM, which causes proteasomal TRF1 degradation [189]. This may occur downstream of ATM phosphorylation by ATR, which is a typical DNA damage response at sites of genomic instability [188,190]. Dissociation of TRF1 would also release the 3′ telomeric overhang from a protective structure and allow telomere elongation by telomerase, which will be discussed in more detail in Section 6. This model is supported by another recent report of ATM-dependent telomerase recruitment in both mice and humans [191], as well as the fact that telomere maintenance defects are observed in ataxia telangiectasia patients (who are deficient in ATM) [192,193]. Inhibition of ATM leads to the shortening of telomeres in mammalian cells and ATM was shown to work with the MRE11-RAD50-NBS1 (MRN) complex to promote telomere elongation by negatively regulating TRF1 association with telomeres [191]. A similar role of posttranslational modification-mediated TRF1 removal from the telomere has been reported for the tankyrase1 protein, a poly(ADP-ribose) polymerase (PARP), which has been reviewed previously by Hsiao and Smith [194]. The many mechanisms behind the recruitment of telomerase seem entwined with controlling telomerase activity at the telomere, which will be the final focus of this review.

## 6. Telomere Maintenance by Telomerase

hTERT binds to the telomere upstream of the hTR template-DNA hybridization region. Following RNA/DNA hybridization at the 3′ telomeric end with the RNA template (which is complementary to approximately two telomeric repeats), the telomerase complex adds the short telomeric repeat sequences. The hTR template is reverse transcribed into DNA by the catalytic activity of hTERT, forming a short telomeric repeat (TTAGGG). The new strand can now be translocated in a 5′ direction for the processive synthesis of additional telomeric repeats on the same telomeric end. It is important to distinguish between telomerase activity, which is its capacity to elongate the telomere by adding a short telomeric repeat at the G-rich single stranded overhang, and its RAP. In 1991, Carol Greider showed that between the two models proposed for telomere maintenance by telomerase, dissociation or processivity, the enzyme is processive [195]. RAP is the unique capacity of hTERT to add multiple telomeric sequences at the same telomeric substrate without completely dissociating. This process is limited by the enzyme’s capacity to translocate and complementarily form DNA/RNA hybridization a few nucleotides downstream of its initial place [196].

### 6.1. hTERT

hTERT contains essential elements for telomerase activity and RAP. The TEN domain of hTERT acts as the anchor site for telomerase to the single stranded DNA, next to the primer-template site [197,198]. It regulates telomerase RAP as this domain is essential for the stability of the RNA/DNA hybrid at the telomere. In fact, TEN domain mutants decrease RNA/DNA hybrid stability and result in failed telomeric primer elongation [199]. Moreover, it was recently shown that various residues located in the IFD of hTERT are critical for telomerase activity and processivity. While some substitutions in this domain (such as V763S) decrease telomerase activity but not RAP, others (such as V791Y and L805A) cause defects in both activity and RAP [180,181]. The impact of the IFD on telomerase activity and processivity is likely due to defective hTERT interaction with the TPP1–POT1 complex. In addition to its aforementioned role in recruitment, TPP1 can regulate telomerase as a processivity factor [200]. The TPP1/POT1 heterodimer increases telomerase processivity through the TPP1 OB domain [173,174,176,201,202]. The TPP1/POT1/hTERT complex is maintained during telomere elongation and translocates on the single stranded telomeric DNA. Interestingly, POT1 can also act as an inhibitor of telomere elongation by binding to the 3′ end of the single stranded overhang and recruiting the CTC1/STN1/TEN1 (CST) fill-in complex, blocking telomerase access to the telomere (also discussed in Section 6.3). Therefore, it is a regulator of telomerase activity and RAP, either by promoting its translocation along the G-rich strand while bound to TPP1 or acting as a “stop” sign at the end of the telomeric single stranded DNA when telomerase is not needed [203,204].

### 6.2. hTR

Many domains of hTR are also essential for the proper elongation of telomeres [205]. The pseudoknot domain contains the template region for DNA synthesis and a pseudoknot structure which marks the end of the template region. Telomerase RNA mutants decrease telomerase activity [109,117] although only its complete deletion can inhibit telomerase recruitment and activity. It has also been observed that the inability of hTR to bind telomeric DNA mostly abolishes telomerase activity [206]. Moriarty et al. demonstrated that hTR template mutants do not directly affect telomerase activity but rather the interactions with specific RNA binding domains of hTERT [207]. In fact, the P1b domain of hTR, which is the template-adjacent stem loop that prevents non-telomeric sequences from being reverse transcribed [208,209,210], is an hTR structural element crucial for telomerase activity. Mutant P1b sequences lead to a decrease in the reverse transcriptase activity of hTERT [211].

Furthermore, the CR4/5 domain is also known as the activation domain and stem terminus element and has been reported to be crucial for telomerase activity. This domain contains elements necessary for the binding of hTERT through the TRBD, as described in Section 3.6. A recent structural study of the medaka telomerase RNA CR4/5 domain suggests that the P6.1 loop in this domain is important for TERT binding [110]. CR4/5 domain mutant RNAs have been linked to DC, as have CR7 domain mutants, containing the H/ACA box discussed in Section 2 [109,110,212]. Most recently, the H/ACA domain of hTR was reported to enhance the endogenous interaction between hTR and hTERT, though hTR lacking the H/ACA domain can yield telomerase activity upon over-expression of hTERT [83]. While hTR and hTERT are critical for telomere elongation, other factors come into play to make the telomere readily available or to remove the enzyme from its substrate. The importance of shelterin-mediated telomerase activity will be discussed next.

### 6.3. The Shelterin Proteins

As demonstrated by TPP1 and POT1 functions, the shelterin proteins can play key roles during telomere elongation by mediating telomerase activity at the telomere. In contrast to the direct enzymatic stimulatory role of TPP1/POT1, TRF1 and TRF2 have been shown to act mostly as negative regulators at the telomere [213]. The silencing of these two shelterin proteins leads to telomere elongation, and their overexpression is negatively correlated with telomere length [213,214]. However, they do not affect telomerase activity directly but seem to affect the available state of the telomeric substrate. Their binding to the double stranded telomeric DNA drives the formation of telomere loops (t-loops), protective secondary telomeric structures blocking the telomerase docking site and telomere elongation (discussed in more detail below). They are found in more abundance on long telomeres and inversely in low levels on short telomeres, driving telomerase preference for shorter telomeres that will be more readily available [215]. However, additional factors associated to these proteins affect telomerase activity. A novel TRF1 associated factor was recently identified. AKT- Interacting Protein (AKTIP) interacts with TRF1 and is recruited at the telomeres. In HeLa cancer cells, AKTIP depletion causes a delay in DNA synthesis [216]. Moreover, AKTIP deletion also results in more fragile telomeres and induces the formation of TIFs (telomere dysfunction induced foci) or telomeric DNA damage, highlighting a role for AKTIP in telomere maintenance. Further experiments would be required to understand if this association factor directly regulates telomerase activity and RAP. Other factors associated with TRF1 are inhibitors of telomerase activity or processivity. PinX1 depletion increases telomerase activity in vivo and the overexpression of its C-terminal domain completely inhibits telomerase activity in telomerase positive cell lines while having no effect in telomerase negative cell lines [217].

TRF2 also recruits related essential factors such as the shelterin component RAP1 and the Apollo exonuclease. RAP1 interacts with TRF2 through its Rap1 C-terminal domain and inhibits telomere elongation in a dosage-dependent manner, presumably through recruitment of an as-of-yet unidentified limiting interacting partner that is required for telomere length regulation. When RAP1 is overexpressed in HCT75 cancer cells, a clear increase of telomere length is observed [218]. This is consistent with the hypothesis that the protein is *cis-*acting in repressing telomere elongation through a limiting interacting factor. When it is overexpressed, the resulting poor telomere length regulation is likely caused by RAP1 accumulation in the nucleoplasm while all TRF2 binding domains at the telomere are occupied, leading to titration of the limiting regulation component from the telomere and subsequent aberrant telomere elongation. A recent study in budding yeast proposes a second mechanism of RAP1 mediated regulation of telomere length. RAP1 might act on internal TG repeats, with the binding of multiple RAP1 proteins inducing double stranded breaks where de novo telomere synthesis can occur [219]. Consistent with a model of RAP1 mediating the DNA damage response at the telomere, Rai et al. also reported that human RAP1 plays an essential role in telomere maintenance by promoting telomere capping when bound to TRF2, thus protecting them from Homology Directed Repair (HDR) [220]. This protein has also recently been shown to associate with (Zinc Finger and SCAN domain containing 4 (Zscan4). Zscan4 interacts through its Zinc finger domain with RAP1 and their expressions are positively correlated [221]. This novel interacting protein seems to increase telomere elongation by inactivating RAP1 and balancing the downregulation of telomerase activity. Still, much is unclear about the specific role of RAP1 in regulating telomere length maintenance, and what distinguishes its repression/activation functions.

The 5′ to 3′ exonuclease Apollo has a role in regulating the length of the 3′ telomeric overhang [222,223,224]. Apollo co-immunoprecipitates with TRF2 and RAP1. Furthermore, knockdown of Apollo leads to the formation of TIFs, mainly in S-phase human cells [224]. Moreover, it is involved in the resection of the 5′ C-rich strand of mouse telomeres, generating the G-rich 3′ overhang needed for telomere protection through t-loop formation [223]. Both Apollo and the 5′ to 3′ exonuclease Exo1 have been reported as resection factors at human telomeres previously [225], involved in the POT1/CST-mediated telomere overhang maintenance mentioned in Section 5. Various other TRF1-TRF2 associated factors have been identified and display regulatory functions in telomerase activity and telomere maintenance such as FLAP Endonuclease 1 (FEN1), Protein Phosphatase 1 Nuclear-Targeting Subunit (PNUTS) and Microcephalin 1 (MCPH1), which have already been thoroughly reviewed [226].

### 6.4. DNA Helicases

For telomerase to act at telomeres, DNA helicases which unwind the secondary and tertiary telomeric structures are essential. At the telomere, extreme nucleic acid structures form to protect the single stranded overhang from being recognized as DNA damage [227], such as G-quadruplexes at G-rich sequences and t-loops which form upon single stranded 3′ overhang invasion into the region of double stranded telomeric DNA. The t-loop thus hides the overhang from a DNA damage repair response. T-loop formation also generates another complex nucleic structure called the D-loop (displacement loop), which is aptly named for the double stranded telomeric DNA that is displaced during single stranded overhang invasion. Two intensive reviews have explored some roles of DNA helicases in telomere elongation through resolving these structures and allowing telomerase access to its substrate [228,229]. Most of these helicases belong to the RecQ family and are canonically involved in genome maintenance by driving the unwinding of DNA in a 3′ to 5′ direction, dependent upon ATPase activity. Mutations in genes encoding these enzymes are associated with predispositions to cancer and premature aging syndromes [230]. This helicase family includes Werner syndrome ATP-dependent helicase (WRN) and Bloom syndrome protein (BLM), both of which are important for telomere maintenance by G-quadruplex unwinding. Loss of function of these proteins has been reported in the premature aging syndromes Werner Syndrome and Bloom Syndrome, respectively [231]. However, WRN loss seems to promote an alternative elongation of telomere pathway which is telomerase-independent [232]. An additional member of this helicase family is Regulator of Telomere Length 1 (RTEL1) which has been reported to actively unwind t-loops [233]. RTEL1 is recruited to the telomere in a TRF2-dependent manner during S-phase through the RTEL1 Cysteine-rich C4C4 motif. It has also been reported to act on G-quadruplexes as well in a proliferating cell nuclear antigen (PCNA)-dependent manner through its PCNA interacting protein box (PIP box) [234]. Many mutations in the *RTEL1* gene have been linked to the severe variant of DC, Hoyeraal-Hreidarsson (HH) syndrome [235,236,237,238,239]. A recent study highlights the importance of a conserved domain in RTEL1 that was previously described by Kuper et al. (2012) [240]. The ARCH domain, which is unique to eukaryotic RTEL1, was characterized with 3D modeling to be in proximity of both the catalytically relevant iron sulfur cluster and the helicase domain at the pore of the helicase. Interestingly, ARCH domain mutants were identified in patients with HH syndrome, emphasizing its importance in RTEL1-mediated telomere maintenance in humans [237]. Further experiments could provide more information on the impact of these mutations on telomere elongation and telomerase activity at the telomere. Other important DNA helicases which have been identified to impact telomere function and have been reviewed in detail include PIF1, FANCJ, and RECQ4 [228,229].

### 6.5. Telomeric Repeat Containing RNA (TERRA)

Another important factor involved in telomerase activity and telomere maintenance is TERRA, a noncoding RNA encoded by subtelomeric DNA and ending in telomeric repeats [241,242]. The idea of TERRA’s inhibitory role on telomerase arose in 2008 with a publication by Schoeftner and Blasco who hypothesized that telomeric RNAs could potentially compete with the telomeric substrate for telomerase interactions. Indeed, this was shown in vitro to be the case for TERRA [241,243]. In fact, a recent study by Azhibek et al. gives the first insight that TERRA has a stronger affinity for hTR than telomeric DNA [244]. Additionally, it is hypothesized that TERRA is not restricted by the formation of telomerase-blocking structures such as t-loops [244]. The transcription of these non-coding RNAs is driven by the critical shortening of telomeres in yeast [245]. In contrast to its hypothesized human role in telomerase inhibition, the higher telomerase affinity for TERRA allows for the specific recruitment of telomerase to shortened telomeres in *S. cerevisiae.* Interestingly, TERRA’s inhibitory role in humans is prevented in vivo. It was suggested that the mechanism behind this is through interaction with hnRNPA1 at the telomere, as it was demonstrated that these two components negate one another’s telomere synthesis inhibitory activities upon their interaction in vitro [246]. It was also shown that TERRA can be recruited to chromosome ends by a TRF2 interaction through the N-terminal glycine/arginine rich (GAR) and myb-type DNA-binding (C-terminal MYB ) domains of TRF2 [247]. More consideration must be given to the true role of TERRA in regulating human telomerase activity at the telomere.

### 6.6. CST Complex

The CST complex which binds to the G-rich single stranded telomeric DNA overhang to cap and regulate telomere length acts as a terminator of telomerase activity. This complex has been reported to inhibit telomerase activity in vitro [248]. Moreover, a recent publication demonstrated that CST function is correlated with POT1 levels at the telomere [249]. A mutated POT1 protein identified in two patients with the autosomal recessive disorder Coats plus (CP) was reported to function canonically with respect to telomeric localization, TPP1 interaction, and protection against inappropriate ATR kinase-mediated DNA damage response at the telomere. However, CP POT1 was unable to negatively regulate telomerase, causing defects in telomere processing and leading to unstable extended 3′ overhangs. This was due to CST depletion at the telomere and uncontrolled telomere elongation. This is similar to what is observed in cells from CP patients with mutant CST complex component CTC1. These patients display a failure to fill-in the 5′ telomeric C-strand and subsequent destabilization of the 3′ overhang, thus suggesting a cooperative role between POT1 and CST in the negative regulation of telomerase recruitment to the telomeres [249]. Therefore, the regulatory mechanism of cooperative POT1/CST telomere elongation is likely in part through fill in synthesis regulation, but more research is needed to fully elucidate telomerase inhibitory roles of CST.

## 7. Conclusions

Regulation of telomerase, and subsequently telomere integrity, is an elaborate cellular process, moderated differently in particular cell types and under varying cellular conditions. The implications of maintaining telomere homeostasis for both cellular aging and cancer underlie the importance of understanding the detailed mechanisms of their maintenance. In this review, we have bridged old and new knowledge of several mechanisms: telomerase assembly, recruitment and activity are necessary regulatory steps for the maintenance of genomic integrity at the telomere. Taken as a whole, many components of telomerase regulation still remain a mystery, though our understanding of each detail in the diverse processes dictating telomerase function is constantly being advanced.

## Abbreviations

The following abbreviations are used in this manuscript:

ALT	Alternative lengthening of telomeres
AAA+ ATPases	ATPases Associated with diverse cellular Activities
AKTIP	AKT-interacting protein
ATM	Ataxia telangiectasia mutated
ATR	Ataxia telangiectasia and Rad3-related protein
BML	Bloom syndrome protein
CAB box	Cajal body box
CB	Cajal body
CBCA	5′ cap-binding complex
CHIP	C-terminus of HSP70 interacting protein
CR4/5	conserved region 4 and 5
CRISPR	Clustered regularly interspaced short palindromic repeats
CRM1	Chromosomal maintenance 1
CST	CTC1/STN1/TEN1
D-loop	Displacement loop
DAT	Dissociates activities of telomerase
DC	Dyskeratosis congenita
DCP2	Decapping mRNA 2
DCP2	decapping mRNA 2
DHS	DNAse I hypersensitive site
ERE	Estrogen response elements
FANCJ	Fanconi anemia group J helicase
FEN1	FLAP enodnuclease 1
H3K9	Histone 3 Lysine 9
Hdm2	Human double minute 2
HDR	Homology Directed Repair
hESC	human embryonic stem cell
HH	Hoyeraal-Hreidarsson
HIF	hypoxia-inducible factor
hnRNPA1	Heterogeneous nuclear ribonucleoprotein A1
HSP90	Heat Shock Protein 90
hTERT	human telomerase reverse transcriptase
hTR	human telomerase RNA
IFD	Insertion in fingers domain
IPF	Idiopathic pulmonary fibrosis
MCPH1	Microcephalin 1
MKRN1	Makorin ring finger protein 1
MRN	MER11-RAD50-NBS1
NEXT	nuclear exosome targeting
NF-KB	Nuclear factor kappa-light-chain-enhancer of activated B cells
NNS	Nrd1-Nab3-Sen1
PARN	polyadenosine-specific ribonuclease
PARP	poly(ADP-ribose) polymerase
PCNA	Proliferating cell nuclear antigen
PINX1	PIN2/TERF1 interacting, telomerase inhibitor 1
PIP box	PCNA interacting protein box
PK	Protein Kinase
Plk1	Polo-like kinase 1
PNUTS	Protein phosphatase 1 nuclear-trafficking subunit
POT1	Protein of telomeres 1
PUA	pseudouridine synthase and archaeosine transglycosylase
RAP	Repeat addition processivity
Rap1	Repressor activator protein 1
RECQL4	RecQ helicase-like 4
RNAPII	RNA polymerase II
RNP	Ribonucleoprotein
RTEL1	Regulator of telomere length 1
S1P	Sphingosin-1-phosphate
scaRNA	small Cajal body specific RNA
SENP1	sentrin-specific peptidase 1
snoRNA	small nucleolar RNA
Sp1	Specificity protein 1
SSD	C-terminal SHQ1-specific domain
SUMO	Small ubiquitin-like modifier
t-loop	telomere loop
TEL	TPP1 glutamate and leucine-rich
TEN	TERT Essential N-terminal domain
TERRA	Telomeric repeat containing RNA
TF	Transcription Factor
TID	Telomerase inhibitory domain
TIF	Telomere dysfunction induced foci
TIN2	TRF1-interacting nuclear factor 2
TPP1	previously known as TINT1 (TIN2 interacting protein), PTOP (POT1 and TIN2 organizing protein) and PIP1 (POT1 interacting protein)
TRAMP	Trf4/5-Air1/2-Mtr4
TRBD	Telomerase RNA binding domain
TRF1/2	Telomere repeat-binding factors ½
Ubc9	Ubiquitin-conjugating enzyme 9
WRN	Werner syndrome ATP-dependent helicase
XPB	Xeroderma pigmentosum type B
Xrn1	5′-3′ Exoribonuclease 1
Zscan4	Zinc finger and SCAN domain containing 4

## Appendix

While the manuscript was under review, a study making use of live cell imaging to observe telomerase trafficking in human cells was published [250]. Schmidt, Zaug, & Cech observe three distinct populations of hTERT during S-phase: a static population of hTERT binds at CBs; a second static population localizes stably at the telomere; and a third highly dynamic population diffuses throughout the nucleus and displays short interactions with chromosome ends as a form of “probing” for telomeres which need elongation. A separation of function mutation in the TEN domain of hTERT, which renders the enzyme fully active but unable to interact with TPP1, abolishes localization of both static and dynamic hTERT populations to the telomere. This study demonstrates the essentiality of the TPP1-hTERT interaction for recruitment of the enzyme to the telomere, in addition to affirming an important role for stable telomerase-CB interactions during S-phase.
